# Down-regulation of miR-30a-5p is Associated with Poor Prognosis and Promotes Chemoresistance of Gemcitabine in Pancreatic Ductal Adenocarcinoma

**DOI:** 10.7150/jca.31191

**Published:** 2019-08-28

**Authors:** Liangjing Zhou, Shengnan Jia, Guoping Ding, Mingjie Zhang, Weihua Yu, Zhengrong Wu, Liping Cao

**Affiliations:** Department of General Surgery, Sir Run Run Shaw Hospital, School of Medicine, Zhejiang University, No. 3, Qingchun Road, Hangzhou, Zhejiang province, China

**Keywords:** miR-30a-5p, FOXD1, prognosis biomarker, pancreatic cancer, gemcitabine, sensitive

## Abstract

MicroRNA-30a-5p (miR-30a-5p) plays an important role in many biological and pathological processes, and therefore has been studied extensively. However, its expression and function in pancreatic ductal adenocarcinoma (PDAC) remain unclear. Furthermore, whether miR-30a-5p affects sensitivity of PDAC cells to gemcitabine (GEM) is worthy of further exploration. The results showed that miR-30a-5p expression in pancreatic cancer was decreased and the down-regulated expression correlated with poor prognosis, while up-regulating miR-30a-5p suppressed tumor cell proliferation, cell cycle and increased apoptosis. MiRNA expression profiles between gemcitabine-resistant pancreatic cancer cells and parental pancreatic cancer cells showed significant change of miR-30a-5p expression. Besides, up-regulating miR-30a-5p in PDAC significantly increased the chemosensitivity of gemcitabine. Furthermore, FOXD1 is a direct target of miR-30a-5p and the miR-30a-5p/FOXD1/ERK axis may play an important role in the development of gemcitabine resistance in pancreatic cancer. In summary, our study showed that miR-30a-5p increases the sensitivity of pancreatic cancer to gemcitabine, and it may be a potential therapeutic target to overcome gemcitabine resistance.

## Introduction

Pancreatic cancer is a common, highly malignant neoplasm with a 5-year survival rate of only 3%-6% 1. It is the fourth leading cause of cancer-related deaths in the United States. Chemotherapy of pancreatic cancer can remarkably improve survival and quality of life for patients with resectable or unresectable pancreatic cancer 2. In 1997, Burris et al demonstrated that gemcitabine significantly improved symptoms and prolonged median survival in a phase III trial 3. Currently, gemcitabine has become the preferred chemotherapeutic drug for advanced pancreatic cancer. However, recent studies have found that a large proportion of patients are resistant to gemcitabine, leading to treatment failure. It was reported that intracellular drug metabolism-related genes (such as hENT1, hCNT1) and apoptosis-related genes (such as IAP families and HMGA1) may affect the therapeutic efficacy of gemcitabine 4-7. However, the exact mechanism of such resistance is still unclear. More gene related to the chemosensitivity of gemcitabine needs to explore.

MiRNAs have been discovered in recent years. They are small, non-coding RNAs with a length of 19-22 nucleotides. These molecules are considered as key regulatory factors for the occurrence and development of tumors. Because miR-30a-5p plays an important role in many biological and pathological processes, it has attracted much attention 8. Li et al found that miR-30a-5p inhibits breast cancer cell proliferation, migration, and invasion by regulating the ERK/Ets-1 signaling pathway 9. In a study by Kumarswamy et al, miR-30a-5p affects the epithelial-mesenchymal transition of cells by acting on the target gene Snail, thereby inhibiting the invasion and metastasis of lung cancer 10. However, its expression and mechanism in pancreatic cancer remain unclear. By searching the Gene Expression Omnibus (GEO) database, we found that expression of miR-30a-5p was low in pancreatic cancer, and its function worth further exploring.

Recent studies indicate that miR-30a-5p is also involved in resistance of chemotherapeutic drugs. Yu et al demonstrated that miR-30a-5p acts as an autophagy inhibitor in chemotherapy of chronic myeloid leukemia, which promotes the chemotherapy-induced cytotoxic response by inhibiting the expression of autophagy genes BECNL and ATG5 11. However, it is unclear whether miR-30a-5p is involved in gemcitabine resistance of pancreatic cancer. We found that expression of miR-30a-5p was significantly down-regulated in gemcitabine-resistant pancreatic cancer cells. Thus, in the present study we focused on the effect and mechanism of miR-30a-5p in promoting the chemosensitivity of gemcitabine in PDAC.

## Materials and Methods

### Cell culture

Human pancreatic cancer cell lines, including Panc-1, BxPC-3, MIAPaCa-2 and normal pancreatic ductal epithelial cell HPDE6-C7 were all purchased from Chinese Academy of Sciences (Shanghai, China) and were cytogenetically tested and authenticated before they were frozen. BxPC-3 cell line was cultured in RPMI 1640 medium containing 10% fetal bovine serum (FBS, Gibco, New York, USA). Panc-1, MIAPaCa-2 and HPDE6-C7 cell lines were cultured in DMEM medium with 10% FBS. For drug treatment, cells were plated into 6-well plates, and 0.4 ug/ml gemcitabine (Eli Lilly and Company. USA) was added to the culture. Cells were collected or tested 48 h after the drug treatment.

### RNA extraction and qRT-PCR

Total RNA of cells was isolated and purified by miRNeasy Mini Kit (Qiagen, Maryland, USA), following the manufacturer's instructions. Reverse transcription (RT) was performed using PrimeScript RT reagent Kit (Takara, Otsu, Japan) following the manufacturer's instructions. The polymerase chain reaction (PCR) primers of miR-30a-5p and U6 used were purchased from Guangzhou RiboBio Co., Ltd. (MQP-0101 and MQP-0202,Guangzhou, China).

### Tissue samples

Formalin-fixed and paraffin-embedded pancreatic ductal adenocarcinoma and adjacent normal pancreatic tissue specimens were obtained from pancreatic cancer patient. The research protocol was reviewed and approved by the Research Ethics Committee of Sir Run Run Shaw Hospital, School of Medicine, Zhejiang University. The study was conducted following the Helsinki Declaration. All participants or their guardians gave written consent of their tissue samples and medical information to be used for scientific research.

### Fluorescence in situ hybridization (FISH)

For the detection of miR-30a-5p expression in tumor sections, fluorescence in situ hybridization assay was performed using the following LNA oligos sequences: LNA-miR-30a-5p 5'-UGUAAACAUCCUCGACUGGAAG-3', which were constructed by GenePharma Corporation (Shanghai, China). Briefly, the formalin-fixed paraffin-embedded tissue sections were deparaffinized in xylene and rehydrated in serial ethanol solutions. The slides were then treated with proteinase K for 20 min at 37°C. Slides were prehybridized in prehybridization buffer at the 78°C for 8 min. After prehybridization, slides were hybridized in hybridization buffer with specific Cy3-labeled probes at 37°C for 16 h. The sections were washed with SSC thoroughly to remove the background signals. Nuclei were counterstained with DAPI, and then sections were analyzed and imaged using a fluorescence microscope (Olympus, Japan).

### Transient transfection

MiR-30a-5p mimics (miR10000087-1-5), FOXD1 siRNA (stQ0001131-1) and their matched negative control (NC) were synthesized by Guangzhou RiboBio Co., Ltd. (Guangzhou, China). Recombinant plasmids over-expressing FOXD1 and matched negative control were constructed by Shanghai Genechem Co.,Ltd (GOCP2301022229). Briefly, 1×10^5^ cells were seeded onto 6-well culture plates. Cell lines were transfected in serum-free medium, transfection was performed using Lipofectamine 3000 following the manufacturer's protocol (Invitrogen). After 24 h of transfection, cells were kept in a culture medium containing 10% FBS up to 48 h. The cells were then harvested and followed by PCR or Western blot analysis.

### Cell proliferation assay

Cell proliferation was measured via CCK-8 assay. Cells were seeded at a density of 5×10^3^/well into 96-well plates and cultured for 72 h. The cells were then incubated with 20 μl CCK-8 for 2 h at 37˚C. The absorbance at 490 nm was recorded.

### Cell apoptosis assays

For cell apoptosis analysis, cells were collected by trypsinization and washed three times using PBS, and then cells were treated with AnnexinV-PE and 7AAD for 30 min according to the instruction manual. Samples were acquired on a BD FACSAria III Cell Sorting System (Becton Dickinson, New York, USA) before analysis using the BD FACSDiva software 6.1.3 (Becton Dickinson).

### Cell cycle analysis

Cells were harvested and washed with PBS, fixed with 75% ethanol overnight at 4℃. The nuclei of the cells were stained with propidium iodide (PI) for 30 min and analyzed with a fluorescence-activated cell sorting caliber system. The results were presented as the percentages of cells in each phase.

### Western blot

Cells were harvested and total protein lysates were resolved using 10% sodium dodecyl sulfate-polyacrylamide gels and electroblotted onto to a polyvinylidene fluoride membrane, blocked by 5% skim milk, and probed with the following antibodies: FOXD1 (1:1000, ab49156, Abcam), ERK (1:1000, 4695, Cell Signaling Technology), p-ERK (1:1000, 4370, Cell Signaling Technology) and GAPDH (1:5000, ab181602, Abcam). The membrane was then incubated with the secondary anti-rabbit secondary antibody and visualized by enhanced chemiluminescence using Kodak X-OMAT LS film (Eastman Kodak, Rochester, USA).

### Animal experiments

PDAC-bearing male nude mice with subcutaneous passage of BxPC-3 were used. When the tumor size reached approximately 5 mm in length, the mice were divided into two groups (3 mice per group) randomly. Stabilized miRNAs (miR-30a-5p agomir and Negative control agomir) were purchased from RiboBio (miR40000087-1-10, Guangzhou, China). MiR-30a-5p agomir (5 nM) or control oligos mixture was injected into the xenografts in a multi-site injection manner every 3 days for two weeks, and then followed by administration with gemcitabine (100 mg/kg). At the end of the 30-day observation period, mice were killed and the tumors were measured. The tumor volume was measured with a caliper, using the formula volume = length × width^2^/2.

### Luciferase assay

The cells were co-transfected with pLMP vectors containing FOXD1 3'UTR and miR-30a-5p mimics. Cells were harvested and subjected to lysis 48 h after transfection. Renilla luciferase activity was used for normalization, and firefly luciferase activity was detected with a dual luciferase reporter assay kit according to the manufacturer's protocol.

### Statistical Analysis

All data were presented as mean ± standard deviation (SD) and analyzed using student's t-test. p value < 0.05 was considered as statistically significance. All data were processed using SPSS, version 19.0 and Graphpad Prism 5.0 software program.

## Results

### Low miR-30a-5p expression in pancreatic cancer cell lines and tissues

**Figure 1:**
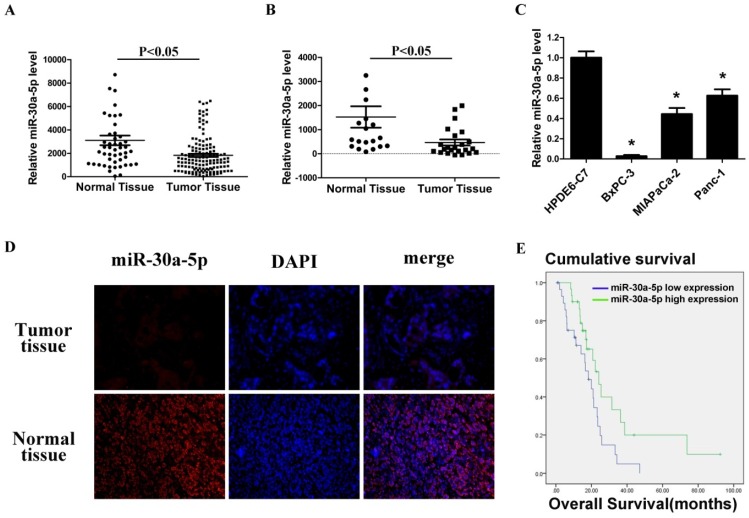
Low miR-30a-5p expression in pancreatic cancer cell lines and tissues. A and B. Relative miR-30a-5p expression level in pancreatic cancer tissues and adjacent normal tissues in a public data set (GSE24279 and GSE29352). C. The expression level of miR-30a-5p in pancreatic cancer cell lines with the normal pancreatic duct epithelial cell line HPDE6-C7 as control. D. The expression level of miR-30a-5p in pancreatic cancer tissue was detected by FISH. E. Effect of the miR-30a-5p expression level on overall survival in pancreatic cancer patients. Data are expressed as mean ± SEM (n = 3). *indicated p<0.05

By analyzing GSE24279 and GSE29352 in the GEO database, we found that miR-30a-5p expression in pancreatic cancer tissues was lower than those in normal paraneoplastic tissues (Figure 1A and 1B, p<0.05). Also, the expression level of miR-30a-5p in pancreatic cancer cell lines BxPC-3, MIAPaCa-2 and Panc-1 was verified lower compared with the normal pancreatic duct epithelial cell line HPDE6-C7 (p<0.05). Among them, the fold change was most significant in BxPC-3 cells (Figure 1C, p<0.05).

Furthermore, 60 cases of pancreatic cancer tissues were collected, and miR-30a-5p expression was detected by fluorescence in situ hybridization. MiR-30a-5p was mainly located in cytoplasm, and the expression of miR-30a-5p in the pancreatic cancer tissues was lower than those in normal pancreatic tissues (Figure 1D). Patients were classified into two groups according to miR-30a-5p expression, and we assessed the potential correlation of miR-30a-5p expression with clinicopathological features and prognosis. We found that decreased expression of miR-30a-5p tended to be poorly differentiated (Table 1, p<0.05). No significant differences were observed according to other clinicopathological features. We also followed up the patients and found that the decreased expression of miR-30a-5p showed a significantly lower survival rate than the high expression group (Figure 1E, p<0.05).

### MiR-30a-5p overexpression inhibits cell proliferation, cell cycle and promotes apoptosis

**Figure 2:**
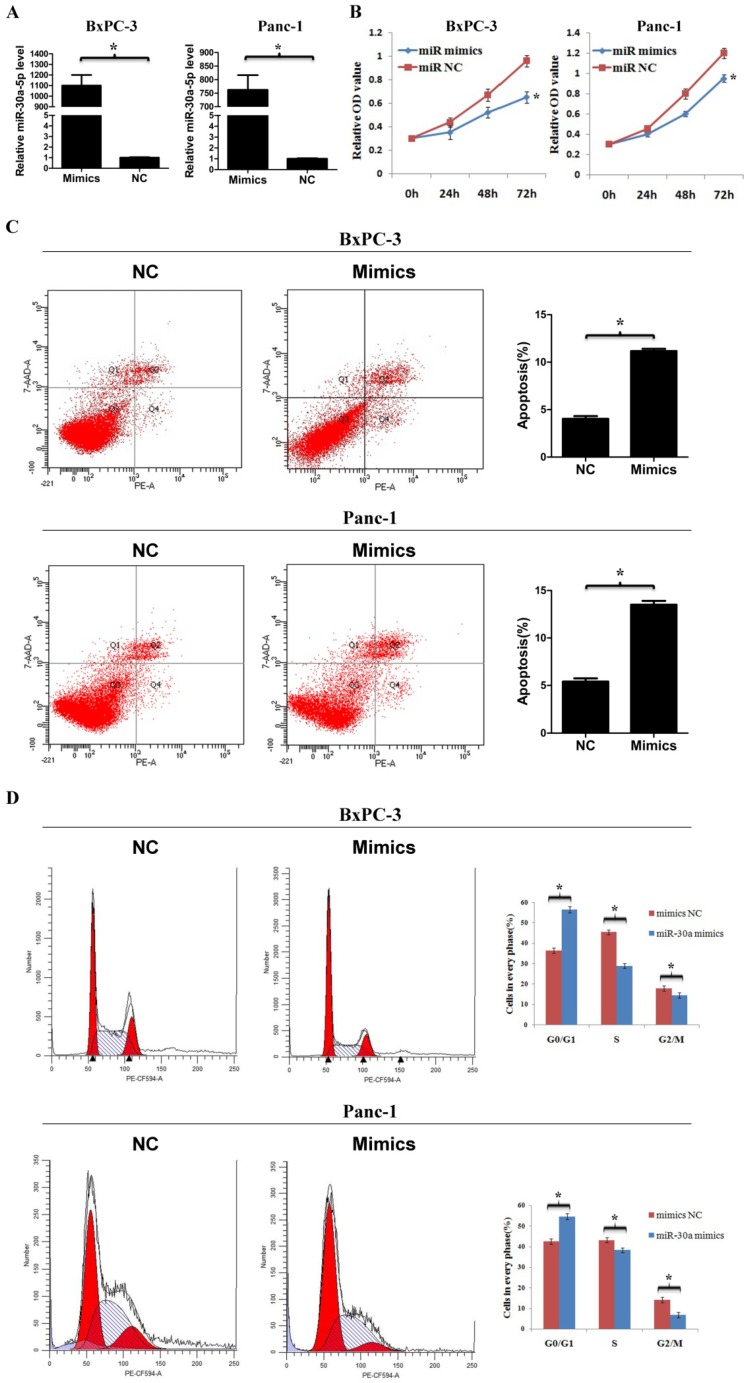
MiR-30a-5p overexpression inhibits cell proliferation, cell cycle and promotes apoptosis. A. MiR-30a-5p was verified over-expressed in BxPC-3 and Panc-1 cell lines by the transfection of miR-30a-5p mimics. B. The CCK-8 assay showed cell proliferation rate decreased significantly after miR-30a-5p overexpression. C. The flow cytometry assay showed the apoptosis level increased significantly after miR-30a-5p overexpression. D. Flow cytometry assay showed the percentage of cells at G0/G1 phase was increased after miR-30a-5p overexpression. Data are expressed as mean ± SEM (n = 3). *indicated p<0.05

To determine the function of miR-30a-5p in pancreatic cancer, we used miR-30a-5p mimics to induce miR-30a-5p overexpression in cell line BxPC-3 and Panc-1 (Figure 2A, p<0.05). The cell counting kit-8 assay showed that the cell proliferation rate was decreased significantly after miR-30a-5p overexpression (Figure 2B, p<0.05). The apoptosis level was increased after miR-30a-5p overexpression (Figure 2C, p<0.05). Moreover, flow cytometry results indicated that the percentage of cells at G0/G1 phase in tumor cells with miR-30a-5p mimics was significantly increased and the proportion at S and G2/M phase was decreased (Figure 2D, p<0.05). Based on these findings, miR-30a-5p overexpression inhibits progression in pancreatic cancer.

### MiR-30a-5p increases the sensitivity of pancreatic cancer to gemcitabine

**Figure 3:**
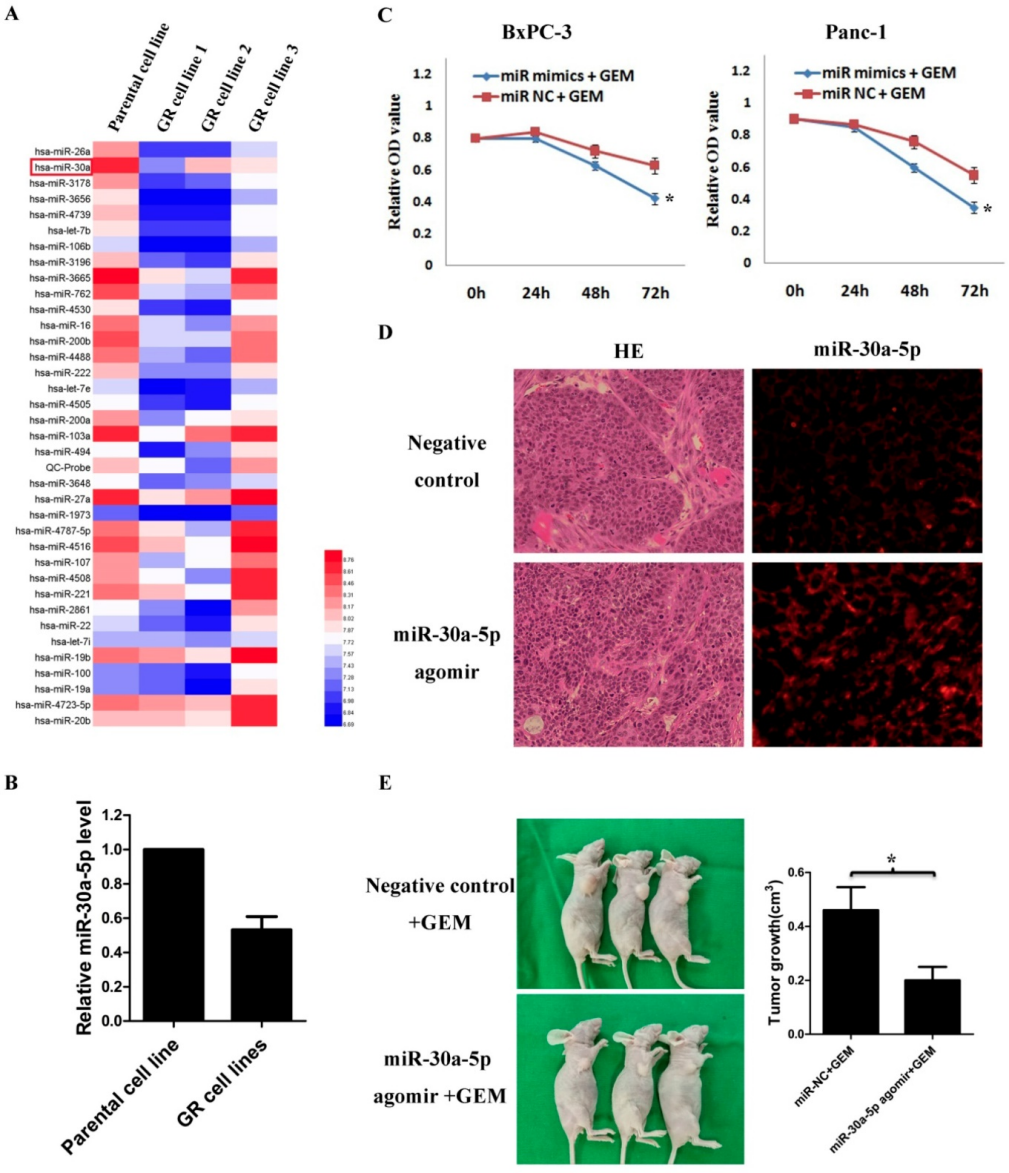
MiR-30a-5p increases the sensitivity of pancreatic cancer to gemcitabine. A. Analysis of the change of miRNAs expression in gemcitabine-resistant pancreatic cancer cell line based on the GEO databases GSE80616. B. Expression of miR-30a-5p in gemcitabine-resistant pancreatic cancer cell line was decreased compared with parental cell line. C. The killing effect of gemcitabine increased in miR-30a-5p over-expressed cells compared to negative control. D. The miR-30a-5p was confirmed increased in the subcutaneous tumors after the intratumoral injection of miR-30a-5p agomir. E. MiR-30a-5p agomir or control oligos mixture (n=3 in each group) was injected into the xenografts in a multi-site injection manner every 3 days for two weeks, and then followed by administration with gemcitabine. Mice were sacrificed at the end of the 30-day observation period and the tumor volume was measured. Data are expressed as mean ± SEM (n = 3). *indicated p<0.05

By analyzing GSE80616 in the GEO database, the differentially expressed miRNAs in gemcitabine-resistant pancreatic cancer cell line were screens out (Figure 3A), and we discovered that the miR-30a-5p expression was significantly decreased (Figure 3B, p<0.05).

Based on the results, we hypothesized that the change of miR-30a-5p expression may affect the chemosensitivity of gemcitabine in pancreatic cancer cells. Pancreatic cancer cells were treated with gemcitabine following miR-30a-5p overexpression, and the killing effect of gemcitabine was increased significantly (Figure 3C, p<0.05). To further confirm the role of miR-30a-5p in vivo, we established xenograft tumor model of PDAC on nude mice. MiR-30a-5p expression was confirmed significantly increased in subcutaneous tumors after intratumoral injection of miR-30a-5p agomir by FISH (Figure 3D). It was observed that the miR-30a-5p over-expressed tumors were obviously smaller than the control ones after they were under the equal dose of gemcitabine treatment (Figure 3E, p<0.05).

### FOXD1 is a direct target for miR-30a-5p

**Figure 4:**
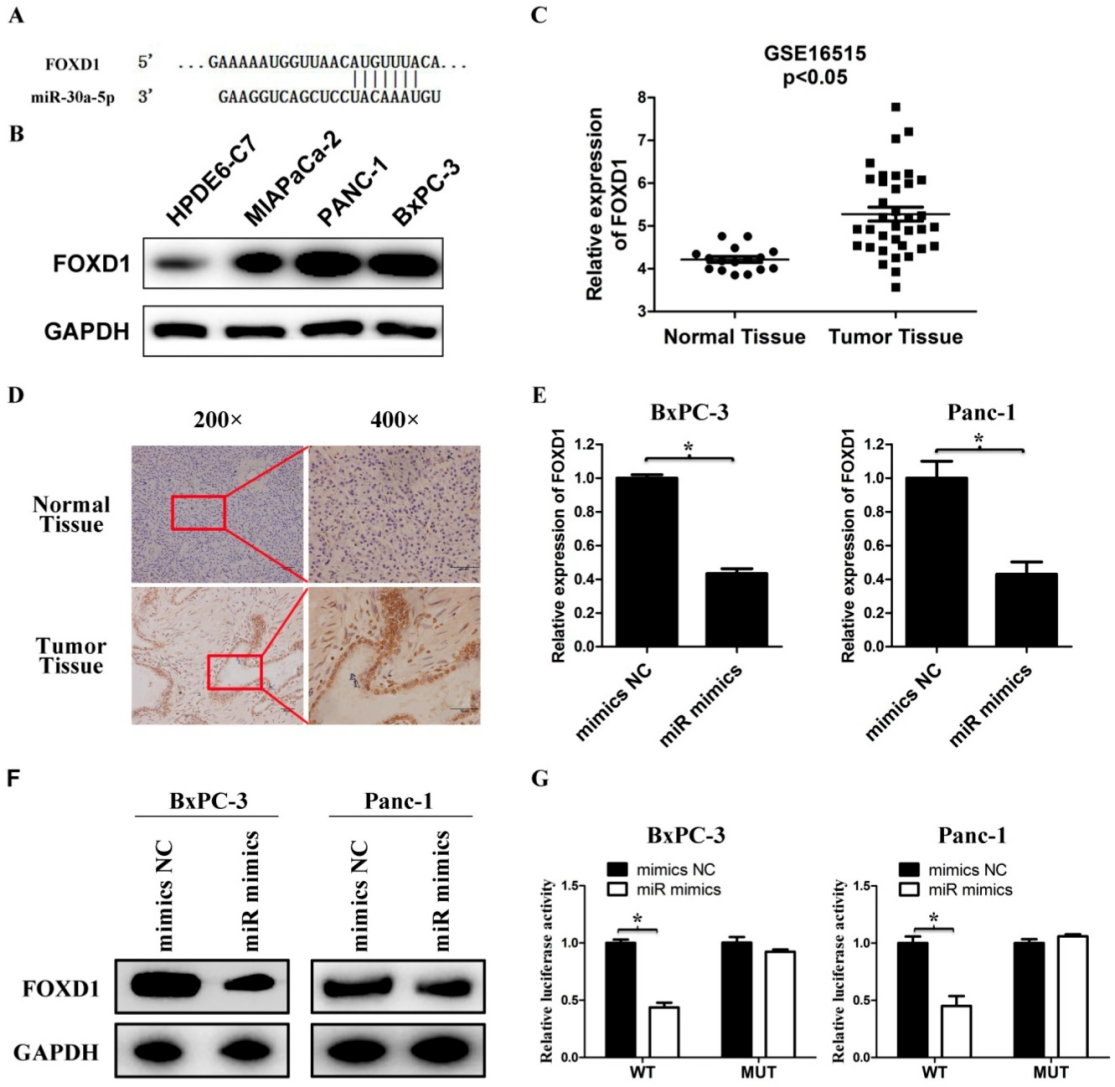
FOXD1 is a direct target for miR-30a-5p. A. The 3' UTR of FOXD1 contains a complementary matching region of miR-30a-5p through predictive analysis by bioinformatics websites. B. The expression level of FOXD1 in pancreatic cancer cell lines with the normal pancreatic duct epithelial cell line HPDE6-C7 as control. C. Relative FOXD1 expression level in pancreatic cancer tissues and adjacent normal tissues in a public data set (GSE16515). D. The expression level of FOXD1 in pancreatic cancer tissue was detected by IHC. E. Expression of FOXD1 in cell lines transfected with miR-30a-5p mimics was detected by qRT-PCR. F. Expression of FOXD1 in cell lines transfected with miR-30a-5p mimics was detected by western blot analysis. G. Luciferase activity of the construct containing the FOXD1 3'-UTR reporter gene or mutant type (MT) FOXD1 3' UTR in cell lines co-transfected with the miR-30a-5p mimics. Relative Renilla luciferase activity was determined and normalized against firefly luciferase activity. Data are expressed as mean ± SEM (n = 3). *indicated p<0.05

We used bioinformatics websites TargetScan and miRDB for predictive analysis. The 3'-UTR of FOXD1 contains a complementary region to miR-30a-5p (Figure 4A). The FOXD1 expression in pancreatic cancer cell lines was verified much higher compared with the normal pancreatic duct epithelial cell line HPDE6-C7 (Figure 4B). By analyzing GSE16515 in the GEO database, FOXD1 expression in pancreatic cancer tissues was higher than those in normal paraneoplastic tissues (Figure 4C, p<0.05). Also FOXD1 expression was detected by IHC and the expression of FOXD1 in the pancreatic cancer tissues was stronger than those in normal pancreatic tissues (Figure 4D). To confirm specific regulation of FOXD1 by miR-30a-5p, we carried out a transfection experiment. Compared with control cells, miR-30a-5p-overexpressing cells had significantly decreased FOXD1 mRNA and protein levels (Figure 4E and 4F, p<0.05). To further assess whether FOXD1 is a direct target of miR-30a-5p, we performed luciferase reporter assays. MiR-30a-5p overexpression significantly decreased activity of the 3′-UTR of the luciferase reporter gene. However, miR-30a-5p exhibited no effect on the pLMP reporters containing mutant type FOXD1 3' UTR (Figure 4G, p<0.05), confirming that miR-30a-5p directly targeted FOXD1 mRNA.

### Overexpression of miR-30a-5p increases the sensitivity of pancreatic cancer to gemcitabine by targeting FOXD1

**Figure 5:**
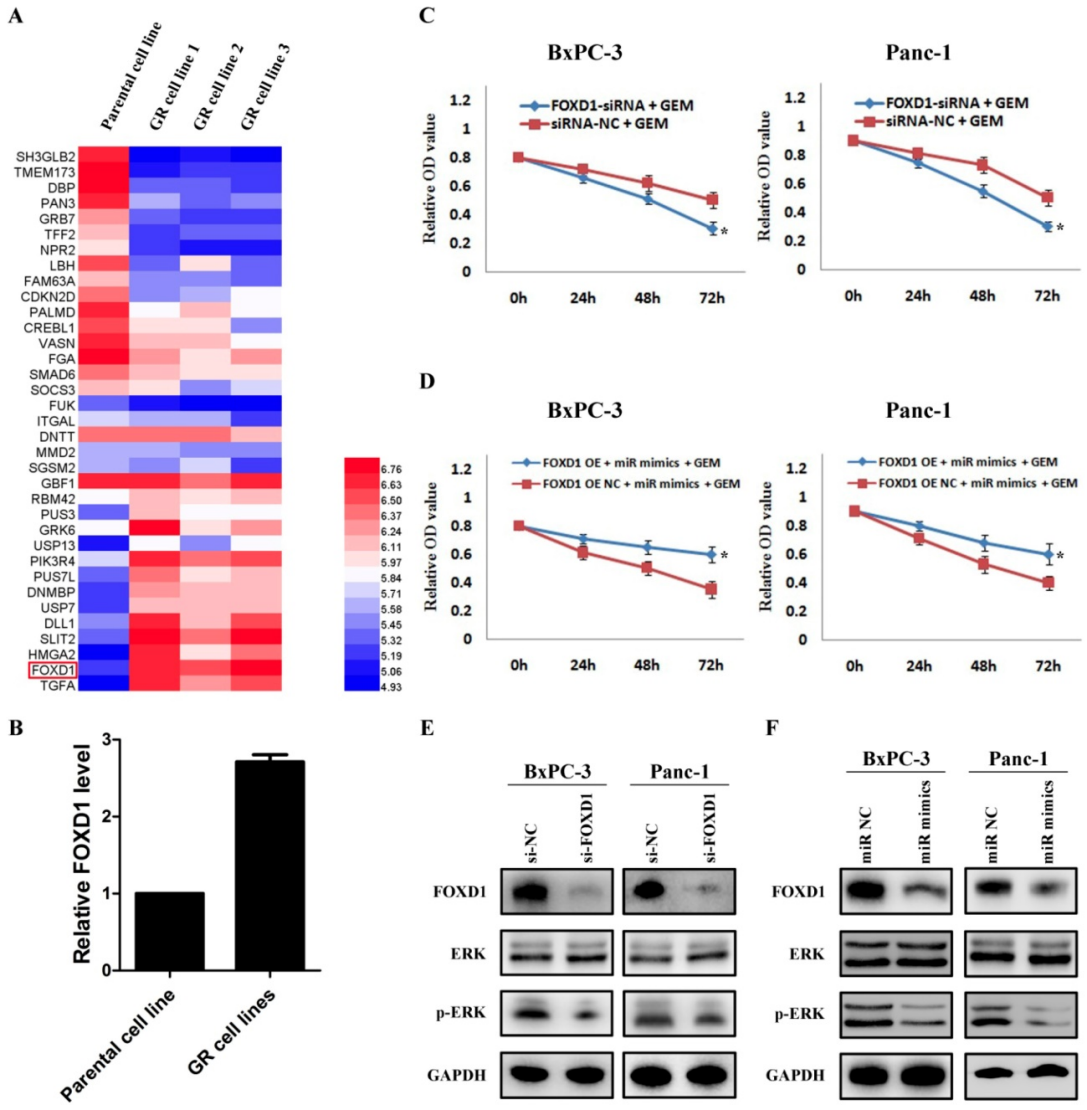
Overexpression of miR-30a-5p increases the sensitivity of pancreatic cancer to gemcitabine by targeting FOXD1. A. Analysis of the change of mRNAs expression in gemcitabine-resistant pancreatic cancer cell line based on the GEO databases GSE80617. B. Expression of FOXD1 in gemcitabine-resistant pancreatic cancer cell line was increased compared with parental cell line. C. The killing effect of gemcitabine increased in FOXD1 knockdown cells compared to negative control. D. FOXD1 overexpression inhibited the effect of miR-30a-5p on increasing the sensitivity of gemcitabine. E. The expression of ERK signaling was detected in FOXD1 knockdown or control cells. F. The expression levels of FOXD1 and ERK were analyzed by western blot in cell lines treated with miR-30a-5p mimics or negative control. Data are expressed as mean ± SEM (n = 3). *indicated p<0.05

By analyzing the mRNA changes of the previous gemcitabine-resistant cell line based on GSE80617 (Figure 5A), it showed that FOXD1 expression was significantly increased (Figure 5B). Based on the direct interaction between miR-30a-5p and FOXD1, we hypothesized whether miR-30a-5p affected the chemosensitivity of gemcitabine via FOXD1. Pancreatic cancer cells were treated with gemcitabine following FOXD1 knockdown, and the killing effect of gemcitabine increased significantly (Figure 5C, p<0.05). Furthermore, when simultaneously over-expressing miR-30a-5p and FOXD1, it showed the up-regulated FOXD1 inhibited the effect of miR-30a-5p in promoting the sensitivity of gemcitabine (Figure 5D). Previous studies showed that activation of FOXD1 may induce the ERK signaling pathway and chemotherapeutic drug resistance 12,13, and we also confirmed that ERK signaling pathway was inhibited after FOXD1 knockdown in pancreatic cancer (Figure 5E). Furthermore, in line with the results in FOXD1-knockdown cells, we found that FOXD1 and p-ERK expression were significantly lower in the miR-30a-5p overexpressing cells compared with controls (Figure 5F). Therefore, the overexpression of miR-30a-5p may increase the sensitivity of pancreatic cancer to gemcitabine by targeting FOXD1.

## Discussion

Recent studies have shown that miRNAs are closely related to the development of tumors. Microarray analyses indicate that miR-30a-5p expression decreases in breast, gastric, and rectal cancer, suggesting that miR-30a-5p may be a tumor suppressor gene 14,15. However, the expression and function of miR-30a-5p in pancreatic cancer have not been reported. In this study, miR-30a-5p expression was also low in pancreatic cancer cell lines and tumor tissues, and over-expressing miR-30a-5p significantly inhibited the cell proliferation, cell cycle and promoted apoptosis. Thus, miR-30a-5p may be a potential therapeutic target.

Recent studies also have found a correlation between low expression of miR-30a-5p and resistance to chemotherapeutic drugs. It was reported that low miR-30a-5p expression in tumors may promote expression of Beclin-1, an autophagy-related protein, thereby promoting the autophagy level and enhancing the efficacy of cisplatin and adriamycin ^16.17^. In addition, Meng et al showed that miR-30a-5p overexpression increases the chemotherapeutic effect of EGFR inhibitor on non-small cell lung cancer by modulating the PI3K/AKT signaling pathway 18. However, no study investigated the role of miR-30a-5p in gemcitabine resistance. Among studies on gemcitabine resistance in pancreatic cancer, it has been reported that cancer genes c-Src and bcl-XL, and the proinflammatory NF-κB signaling pathway are closely related to gemcitabine resistance 19-21. However, the underlying mechanisms are complex and require further investigations. Based on the present study, it showed miR-30a-5p overexpression significantly enhanced the chemosensitivity of gemcitabine to pancreatic cancer cells by directly targeting FOXD1.

FOXD1 is a newly discovered member of the FOX transcription factor family, which is involved in a number of biological processes 22. It has been considered as an oncogene and contributes to proliferation in many types of cancers. Liu et al reported that FOXD1 knockdown leads to inhibition of cell migration and proliferation in glioma 23. Furthermore, Su et al reported that FOXD1 promotes chemoresistance by targeting p27 in breast cancer 12, which means FOXD1 also plays an important role in chemotherapeutic drug resistance. It was firstly reported by Wang et al that miR-30a-5p targets FOXD1 gene in osteosarcoma cell 24. Consistent with the previous finding, in the present study too, we elucidated that the miR-30a-5p/FOXD1 axis is one of the mechanisms that regulates the chemosensitivity of gemcitabine in PDAC, and interfering with miR-30a-5p/FOXD1 axis might be a therapeutic strategy for pancreatic cancer.

In summary, the present study showed that expression of miR-30a-5p was low in pancreatic cancer, and overexpression of miR-30a-5p inhibited cell proliferation, cell cycle and promoted apoptosis. In addition, study showed that overexpression of miR-30a-5p increased chemosensitivity of pancreatic cancer cells to gemcitabine. FOXD1 is a direct target of miR-30a-5p, and the miR-30a-5p/FOXD1/ERK axis played an important role in the development of gemcitabine resistance in pancreatic cancer. In future tailored therapies, miR-30a-5p may be a potential therapeutic target to overcome gemcitabine resistance in pancreatic cancer patients.

## Figures and Tables

**Table 1:** Relationship between miR-30a-5p expression and clinicopathological features

Parameters	miR-30a-5p expression		P values	
	Low	High		
Age				
<60	11	7	0.26	
≥60	19	23		
Gender				
Male	15	16	0.796	
Female	15	14		
Smoking history				
Yes	23	25	0.519	
No	7	5		
Tumor site				
Head	22	25	0.347	
Body + Tail	8	5		
Pathological grade				
Poor and Middle	25	18	0.045*	
High	5	12		
Tumor size				
≤4cm	21	20	0.781	
>4cm	9	10		
T stage				
T1-2	7	4	0.317	
T3-4	23	26		
Lymph node metastasis				
No	9	10	0.781	
Yes	21	20		
Distant metastasis				
M0	28	28	1.000	
M1	2	2		
TNM stage				
I-II	10	7	0.486	
III -IV	20	21		

* P<0.05
